# Supplementary material for: A whole-animal phenotypic drug screen identifies suppressors of atherogenic lipoproteins
Source: eLife. 2026 Aug 27;14:RP105314. doi: 10.7554/eLife.105314 (PMC13521566; doi:10.7554/eLife.105314)
Supplement: Supplementary file 2 [file elife-105314-code1.zip › JHDL_primary_result/FREQUENTIN/FREQUENTIN_JHDL_3_040_007_2019_04_26_14_14.pdf]

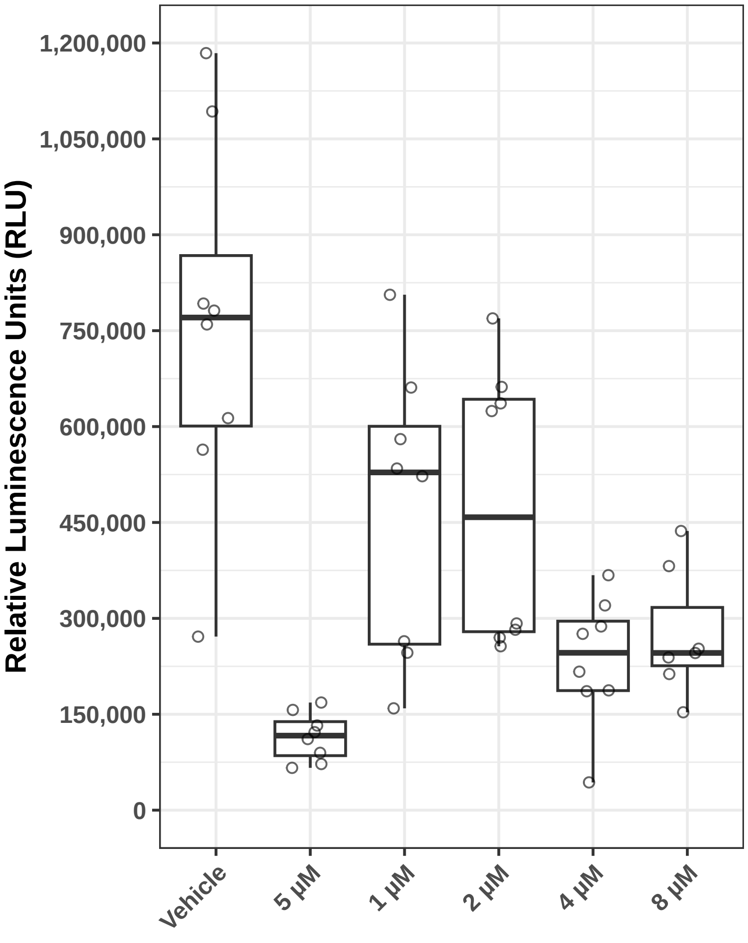

Relative Luminescence Units (RLU)
1,200,000
1,050,000
900,000
750,000
600,000
450,000
300,000
150,000
0
Vehicle
5 μM
1 μM
2 μM
4 μM
8 μM
